# Balancing Promise and Peril: Hemophilia Gene Therapy Insights

**DOI:** 10.1002/iub.70087

**Published:** 2026-01-19

**Authors:** Saicharan Akula, Ester Borroni, Alessia Cottonaro, Antonia Follenzi, Simone Merlin

**Affiliations:** ^1^ Department of Health Sciences Università degli Studi del Piemonte Orientale Novara Italy

## Abstract

Hemophilia is an inherited disorder characterized by impaired blood clotting caused by mutations in the genes responsible for producing coagulation factor (F) VIII (hemophilia A, HA) or FIX (hemophilia B, HB). Current treatment primarily relies on replacement therapy, involving frequent and costly infusions of FVIII or FIX concentrates. While effective, these treatments come with the risk of developing neutralizing antibodies (inhibitors) against the infused factor. In recent years, non‐factor replacement therapies have emerged as innovative treatment options, offering enhanced efficacy especially for patients with inhibitors. Despite their advantages, these approaches still fall short of providing a definitive, long‐term cure. Since hemophilia is a monogenic disease, it presents an excellent opportunity for cell and gene therapy approaches aimed at achieving durable treatment and potentially a cure. Over the past three decades, remarkable advancements have been made in hemophilia gene therapy, culminating in the approval of Valoctocogene roxaparvovec (ROCTAVIAN, AAV‐FVIII) and Etranacogene dezaparvovec (HEMGENIX, AAV‐FIX) for patients with severe HA and HB, respectively. Nevertheless, gene therapy poses questions regarding its long‐term efficacy and safety. This review synthesizes findings from clinical trials, addresses persistent challenges in hemophilia gene therapy, and underscores the biological constraints and limitations inherent to viral vector‐based approaches.

## 1 Introduction

Hemophilia is an inherited blood disorder that arises from the absence or reduced activity of coagulation FVIII, causing hemophilia A (HA) or FIX, leading to hemophilia B (HB) respectively. As X‐linked genetic diseases, HA and HB predominantly affect males, with an incidence of one male in every 5000–10,000 births for HA and one in 30,000 male births for HB [1]. Numerous mutations have been identified in FVIII and FIX, with 6211 and 1692 variants reported in gene databases (www.factorviii‐db.org/ and www.factorix.org/) as of February 2025. These mutations result in varying levels of residual factor activity, allowing hemophilic patients to be classified as severe (< 1% activity), moderate (1%–5% activity), and mild (5%–40% activity). Due to their impaired coagulation ability, hemophilia patients experience recurrent spontaneous bleeding episodes, eventually resulting in chronic damage to soft tissues, joints, and skeletal muscles. The clinical hallmark of hemophilia is intra‐articular bleeding (hemarthrosis) predominantly in the knee, elbow, and ankle joints, leading to the development of painful and debilitating arthropathies [2]. Since no definitive cure exists, hemophilia patients manage their bleeding events with frequent intravenous infusions of the missing factor as prophylaxis. This approach maintains plasma levels of the infused factor ≥ 3%–5% to prevent spontaneous bleeding [3]. Notably, starting this treatment at an early age showed the highest efficacy in preventing the development of arthropathy caused by hemarthrosis [4]. However, replacement therapy has significant drawbacks. The relatively short half‐life of the infused factors (8–18 h for FVIII and 18–100 h for FIX) compels hemophilic patients to undergo frequent factor infusions (2–3 times per week) [5], even though newer extended half‐life (EHL) factors, such as Altuviiio (efanesoctocog alfa) for FVIII and Idelvion (albutrepenonacog alfa) for FIX, offer significantly longer stability, allowing for once‐weekly dosing [6, 7]. Additionally, these treatments can also lead to the development of neutralizing antibodies in approximately 30% of severe HA patients and 3% of all HB patients [8, 9, 10].

**FIGURE 1:**
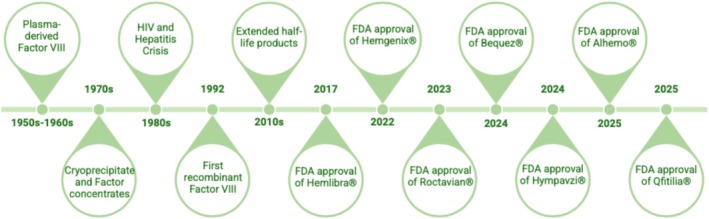
Timeline illustrates the milestones in Hemophilia treatment history, from the discovery of Factor VIII in the 1950s to the latest FDA‐approved gene therapies.

Recent advances in non‐factor replacement therapies have introduced innovative alternatives for hemophilia management. These include novel substitution therapies, such as bispecific antibodies like Emicizumab (Hemlibra) and Mim8 for HA, and rebalancing therapies to enhance hemostasis, such as RNA interference Fitusiran (Qfitilia) and tissue factor pathway inhibitor Marstacimab (Hympavzi) and Concizumab (Alhemo) [11, 12, 13, 14] (Figure 1). These methods are particularly effective in treating inhibitor‐positive patients.

Despite these advancements, current therapeutic approaches do not fully address the bleeding phenotype. Patients may still require factor concentrates in situations of severe bleeding, trauma, or surgery. Consequently, research is focusing on new cutting‐edge methods, like cell and/or gene therapy, that can guarantee a stable, long‐term correction of the hemorrhagic phenotype of patients, pointing at higher FVIII or FIX activity levels than current therapies, avoiding the immune response to the missing factor and significantly improving patients' quality of life.

In this review we discuss the existing challenges related to hemophilia gene therapy, addressing limitations related to transgene expression, cell/tissue targeting, dosage, immune responses, and scalability of viral vector production and briefly introducing progresses to overcome these limitations.

## 2 Gene Therapy for Hemophilia

Over the past four decades, significant progress has been made in the treatment of HA and HB, marking pivotal moments in the history of hemophilia care (Figure 1). Among these advancements, the introduction of gene therapy stands out as a transformative breakthrough.

The pursuit for gene therapy for hemophilia began in the 1990s, driven by the disease's distinct genetic basis, well‐defined clinical endpoints, and the availability of animal models. Foundational proof‐of‐concept studies demonstrated that adeno‐associated viral vectors (AAVs) could sustain long‐term expression of clotting factors in canine and murine models, laying the groundwork for human translation [15].

Clinical evidence shows that even modest increases in plasma coagulation factor levels—ranging from 1% to 5%—can markedly reduce bleeding risk in patients with severe hemophilia [3]. This therapeutic threshold, achievable despite imprecise gene regulation, positions hemophilia as an ideal candidate for gene therapy. Accordingly, the primary challenge is not insufficient expression, but rather the prevention of transgene overexpression, which may elevate coagulation factor levels and trigger thrombosis—a potentially serious adverse event [16].

**FIGURE 2:**
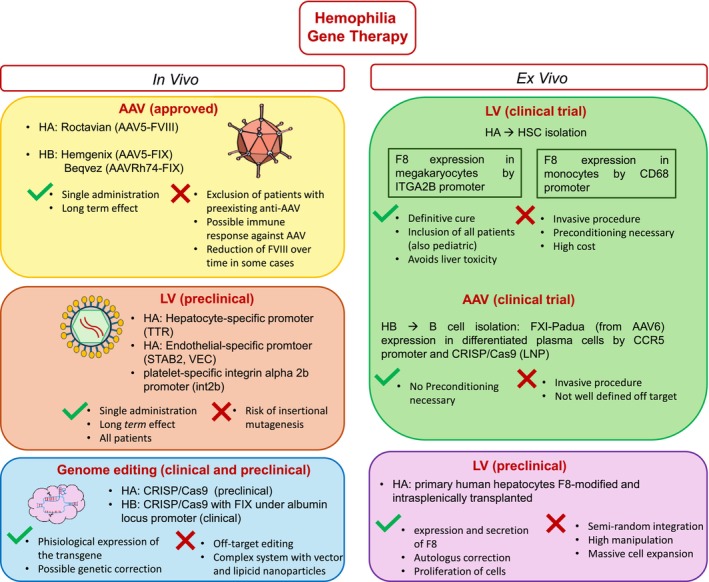
Hemophilia gene therapy approaches approved and currently used in clinical and preclinical studies.

Three gene therapy approaches for hemophilia are currently under investigation (Figure 2): (i) in vivo gene therapy, with direct infusion of a vector carrying the therapeutic gene; (ii) ex vivo gene therapy, which entails the transplantation of gene‐modified cells to enable transgene expression; (iii) gene editing, which uses nucleases to precisely correct the deletions or the mutations at their original location, or to facilitate the integration of a therapeutic gene sequence (whole or partial) into a specific site in the genome, thus ensuring stable production of the missing factor.

Among the viral vectors utilized in gene therapy, two of them are most used in both preclinical and clinical investigations. In vivo gene therapy is typically carried out using adeno‐associated viral vectors (AAV) [17], while ex vivo gene transfer into hematopoietic and other stem cells is achieved by lentiviral vectors (LV) [18].

AAV are recombinant viral vectors with a cargo capacity of roughly 4.7 kb, mostly used in clinical settings/trials for the treatment of monogenic disease, and are the preferred vectors for in vivo gene therapy since they are not associated with any symptom or disease [19]. Additionally, they are considered to have a superior safety profile since their non‐integrating nature allows long‐term transgene expression in episomal form in both dividing and non‐dividing cells. On the contrary, LV can integrate the therapeutic transgene cassette into the genome of both dividing and non‐dividing cells. This unique capability makes LV a versatile tool for gene delivery. Additionally, LV offers a greater cargo capacity of up to approximately 7 kilobases, exceeding the limitations of AAV in terms of transgene size [20].

Several hemophilia gene therapy approaches have been investigated and developed over the past three decades, but the most cutting‐edge ones use systemic administration of recombinant AAV containing the sequence of F8 or F9 codon‐optimized variant transgenes under the control of a liver‐specific promoter. All these results from preclinical studies and clinical trials led to the approval of Etranacogene dezaparvovec (HEMGENIX, AAV‐FIX) and Valoctocogene roxaparvovec (ROCTAVIAN, AAV‐FVIII) in 2022 and 2023 by the US Food and Drug Administration (FDA) and by the European Medicines Agency (EMA) for the treatment of severe HB and HA patients. One of the most important challenges for the efficacy of in vivo AAV gene therapy is the issue of anti‐AAV neutralizing antibodies (NAbs) that the immune system produces against AAV. Since AAVs are very common nonpathogenic viruses for humans, many individuals have already developed these antibodies following subclinical infections at some point in their lives. For this reason, 30%–60% of people are currently ineligible for AAV‐based gene therapy due to pre‐existing immunity toward AAV responsible for the variable presence in the world population of anti‐AAV antibodies capable of neutralizing these viral vectors. This percentage in the world population of NAbs varies according to several factors, such as AAV serotype spreading, geographical location, and age [21, 22].

Ex vivo gene transfer is a gene therapy approach in which patient‐derived cells are genetically modified outside the body and subsequently re‐infused, offering the advantage of controlled gene delivery and safety validation before administration [23]. A recent study demonstrated an ex vivo gene therapy approach in which human hepatocytes were genetically modified to express coagulation factor VIII (FVIII) and subsequently transplanted into immunocompromised hemophilia A (HA) mice, resulting in therapeutic benefits and offering a potential treatment strategy for HA [24].

Due to their low immunogenicity and immunomodulatory impact, several target cell types, such as mesenchymal stem cells isolated from adipose tissue, bone marrow, umbilical cord, and placenta, present high clinical application potential [25].

In this context, induced pluripotent stem cells (iPSCs) are also used in preclinical studies and clinical trials [26, 27] since they exhibit traits resembling those of embryonic cells without the bioethical issues that go along with them. Significant results in the fields of ex vivo cell and gene therapy for HA and HB preclinical studies have been obtained using iPSCs [28, 29, 30] and more recently blood outgrowth endothelial cells (BOECs) [31].

Preclinical studies of HA [32, 33, 34] and limited clinical observations [35, 36] showed that hematopoietic cells can produce FVIII; therefore, suggesting that hematopoietic cells, including hematopoietic stem and progenitor cells (HSPC), can be exploited in HA cell and gene therapy approaches.

Recently, ongoing clinical trials for HA exploiting LV‐FVIII‐transduced autologous HSPC (NTC03818763 and NTC05265767) published results from treated patients, showing the efficacy of this approach [37, 38]. Moreover, this approach represents a novel possibility to treat pediatric patients. However, these studies are preliminary and, to date, only a few patients have been enrolled; thus, more participants, additional clinical trials, and longer follow‐ups will be required to understand their real effectiveness and to understand whether this can become a universal approach.

Recent advances in *genome editing* have opened new avenues for hemophilia gene therapy. Initial efforts used zinc‐finger nucleases (ZFNs) to insert the FIX gene into the albumin locus, but this strategy did not achieve therapeutic expression in clinical trials [39] despite the promising results shown in preclinical studies [40]. Following these early efforts, the field turned to the CRISPR‐Cas9 technology with an approach using a dual‐vector system, rAAV8 and lipid nanoparticles (LNP), that allowed efficient targeted insertion and therapeutic levels of FIX expression in HB preclinical models [41, 42]. This approach is currently used in an ongoing phase 1/2 clinical study with severe and moderately severe HB patients (REGV131‐LNP1265; NCT06379789). A similar dual‐vector approach has shown success in preclinical studies for hemophilia A using a novel nuclease, while other approaches are exploring non‐viral (LNP‐only) vectors [43] and ex vivo cell therapies that edit B cells by introducing the FIX gene within the C‐C chemokine receptor type 5 (CCR5) locus. For this approach, a Phase 1/2 clinical study (BeCoMe‐9) is planned to evaluate its safety and efficacy in adults with moderate to severe HB FIX [44].

## 3 Limitations of Hemophilia Gene Therapy

Although hemophilia gene therapy has recently made significant progress in the clinical setting, with FDA and EMA approval of AAV‐based gene therapy products for both HA and HB, these novel approaches still face important challenges that are currently the focus of several studies aimed at improving therapeutic outcomes and expanding treatment access to all the patients.

One of the primary limitations, particularly for HA, lies in transgene expression. Early studies in hemophilia gene therapy were performed in the HB model and by the F9 transgene, which is small in size (~1.5 kb) and can be easily inserted into different viral vectors, including the one with lower cargo capacity as AAV [45]. Owing to these advantages, the first clinical trial for HB gene therapy using AAV was published as early as 2011 [46].

Similar clinical trials for HA were delayed because of the bigger F8 transgene size, which is approximately 4–5 times higher compared to F9, thus exceeding the ~4.7 kb AAV capacity, and showed a reduced level of expression [47]. Several strategies were then adopted to improve the usage of FVIII transgene, starting from the removal of the B domain that, despite representing roughly 40% of the protein, is not required for the clotting activity [48], the replacement of the B domain with the insertion of a 14 aa link SQ that improves intracellular cleavage of the primary single chain FVIII [49], and the codon optimization which leads FVIII expression profile up to 10‐fold [50].

Despite the higher expression profile of FIX compared to FVIII, in the first clinical trials for HB, transgene expression was ≤ 5% [51]; changing the FIX transgene with a naturally occurring mutated form, FIX‐R338L (FIX‐Padua), resulted in significant improvements, with 8‐fold enhanced activity [52]. Patients treated with an intermediate AAV dosage (5 × 10^11^ vg/kg) demonstrated up to 10‐fold higher FIX activity compared to previous findings [53].

A second limitation is represented by the target cell/tissue, which involves not only the choice of the transgene but also the vector for gene transfer and the expression system. Selecting the right AAV serotype/pseudotype and/or the best combination of transcriptional and post‐transcriptional sequences regulating transgene expression in the target cell/tissue contributes to the success of the gene therapy [54, 55, 56, 57, 58]. Targeting the correct cell/tissue type with AAV requires choosing a specific serotype, for which however pre‐immunity may be more frequent in the general population [21, 22]. Additionally, the target cell type/tissue for transgene expression plays a major role. For example, the target cells for FVIII production in recent trials are the hepatocytes, which are not the native cells for endogenous FVIII synthesis. While clinical studies on HB have shown stable transgene expression for up to 13 years with a single vector administration [59] and despite the stable results obtained in HA dogs [60], AAV‐mediated HA gene therapy clinical trials, even though they showed successful results, exhibited a consistent year‐over‐year decline in FVIII activity levels [61, 62].

LVs for in vivo hepatic gene therapy have been engineered to confine transgene expression specifically to hepatocytes by combining transcriptional regulation via hepatocyte‐specific promoters with post‐transcriptional control through microRNA target sequences [63]. In preclinical models, these vectors have demonstrated stable and sustained long‐term expression of the therapeutic protein directly within the liver [64, 65, 66]. Despite these promising results, the integrating nature of LVs remains a major barrier to their direct in vivo application in clinical trials due to concerns over insertional mutagenesis and long‐term genomic safety.

The liver is responsible for numerous biological functions, including protein synthesis, and for this reason it is highly susceptible to endoplasmic reticulum (ER) stress. In the case of liver‐directed HA gene therapy, FVIII overexpression in hepatocytes could be responsible for inducing cellular ER, thus resulting in loss of transduced cells as observed in a mouse model of HA [67, 68, 69]. The primary source of FVIII in the body is represented by endothelial cells, mainly liver sinusoidal endothelial cells (LSEC), and to a lesser extent hematopoietic cells, as demonstrated by several studies in the last decade [32, 33, 34, 70, 71, 72]. These cell and/or gene therapy preclinical studies showed that by targeting naturally FVIII‐producing cells, it is possible to achieve not only a sustained long‐term transgene expression, thus treating the bleeding phenotype, but also avoid immune responses to the FVIII, hence paving the way for new therapeutic approaches [57, 58].

Immunity is another key limitation for gene therapy. While pre‐existing immunity against LV is virtually absent in the human population, pre‐existing NAbs have a different distribution among populations and ages worldwide [21, 22], and even low‐titer NAbs can significantly impact the outcome of the gene therapy [73]. New approaches to reduce the titer of pre‐existing NAbs are under investigation, such as immunoadsorption procedure and administration of Imlifidase (IdeS), an immunoglobulin G–degrading enzyme of *Streptococcus pyogenes* , to remove or reduce NAbs titer before AAV infusion [74]. Detection of NAbs is not standardized, and results can vary according to the methodology used for their determination. This became a crucial point since animal studies and clinical trials for HB showed a non‐consistent association between NAbs titer affecting the gene therapy results and vector dosage [73], thus requesting new data from preclinical and clinical studies able to establish a cut‐off for the NAbs titer to increase the number of possible patients to be treated. In addition to pre‐existing immunity against viral vectors, another limitation is represented by inhibitors. Generally, participants enrolled in the clinical trials are only inhibitor‐negative patients (> 100–150 exposure days to replacement therapy), thus excluding individuals with a history of inhibitors. Additionally, it is still unknown whether previously untreated patients (PUPs) will develop inhibitors following gene therapy [67].

Clinical research is increasingly investigating the potential of gene therapy to induce immune tolerance in hemophilia patients who have developed inhibitors, but the trials that led to the drug's approval did not currently include these patients. Gene therapy is proposed as a more effective alternative to conventional immune tolerance induction (ITI) protocols, as the continuous endogenous production of clotting factor may better promote immune desensitization compared to intermittent infusions within the patient's body [75].

Initial studies on tolerance induction via gene therapy were performed in a mouse model of hemophilia B, demonstrating that gene therapy has the potential to induce tolerance and prevent the formation of FIX inhibitors [76, 77]. More recently, the first human evidence of immune tolerance induction via liver‐directed AAV gene therapy was reported: in a patient with hemophilia A, gene therapy successfully eliminated a pre‐existing inhibitor. This landmark finding suggests that AAV‐mediated hepatic gene transfer not only restores clotting factor expression but may also reprogram the immune system to tolerate the transgene product [78, 79].

A possible approach to treat inhibitor positive patients is to “hide” the factor produced by the blood circulation, as recently shown by a clinical study reporting the results after the first 6 month follow up of HA patients with inhibitor history treated with autologous CD34+ cells transduced with a LV containing the FVIII transgene under the control of the platelet‐specific integrin subunit alpha 2b (ITGA2B) promoter (NCT03818763) [38]. This strategy allows the expression and the storage of FVIII into platelet α‐granules, thus preventing its interaction with circulating inhibitors and enabling its release following platelets activation. Despite being preliminary, these outcomes show the feasibility of the approach; however, additional data and longer follow‐ups are required to establish the long‐term efficacy and safety of this treatment.

The problem of immunity leads to further limitation represented by the administration of in vivo gene therapy considering the delivery method, vector dosage and reinfusions. As mentioned above, the presence of NAbs can influence the outcome of the in vivo gene transfer as the formation of antibodies against the viral particles is inevitably generated. Furthermore, these antibodies are capable of cross‐reacting even partially with other serotypes, but enough to compromise the success of subsequent infusion(s) of AAV [45]. The route of administration plays an important role in gene therapy: systemic delivery of viral vectors is more prone to induce the formation of NAbs, thus making further possible reinfusions difficult, while with specialized delivery strategies it is possible to administer the AAV in the desired target tissue/organ with greater precision and efficiency, less dispersion of viral vector into circulation and with a lower vector dosage compared to intravenous injection [80, 81]. Additionally, the dosage used for the AAV‐mediated gene therapy should be considered. During gene therapy studies for other diseases, immune responses against viral capsid proteins or transgene products have been reported [82]. Initially it was thought that AAVs only gave rise to reduced/contained responses by the innate immune system. However, several studies have shown that following administration of high doses of vector, they give rise to responses of the innate and adaptive immune system, with the formation of antibodies, both against AAV and the transgene product, activation of a cytotoxic T cell response, which eliminates the transduced cells and complement activation, resulting in a series of significant side effects, along with ER stress in hepatocytes and/or higher rate of increase in liver enzyme levels (transaminitis) managed with steroids to blunt immune reactions and avoid loss of transduced cells [82, 83, 84, 85].

Finally, a substantial limitation is represented by the platform design and the scalability of the viral vector production. Many efforts are now focused on improving production and achieving scalability, both for AAVs and LVs, that is economically sustainable and guarantees reproducibility in terms of yield and purity. Impurities present in the produced viral vector, such as proteins derived from the producer cells, empty viral particles and contamination from plasmid DNA and helper viruses, can reduce the transduction efficiency of the final viral vectors and activate an innate immune response. For large‐scale production of viral vectors, inducible stable producer cell lines and constitutive packaging cell lines have been developed, and they present advantages over the classical production methods in terms of cost, reproducibility, purity and scalability. However, this approach is complicated by the design of the platform, i.e., different serotypes or pseudo‐typing envelope protein and packaging genes combinations, thus hampering the standardization, and consequently the scalability, of viral vector production [20].

## 4 Concerns Related to Hemophilia Gene Therapy

Despite its limitations and associated concerns, gene therapy has undergone remarkable advancements over the past three decades. Both in vivo and ex vivo gene therapy approaches have demonstrated their efficacy in providing sustained, long‐term treatments or potential cures for a wide range of conditions—including hereditary and acquired hemophilia [86]. However, alongside these significant successes, gene therapy has also faced notable failures and challenges. These issues, particularly related to the biology of viral vectors, remain a source of concern—not only in the context of hemophilia gene therapy but across the entire field of gene therapy.

For the treatment/cure of hereditary blood diseases, it is possible to opt for HSPC transplant from a compatible healthy donor (allogeneic), or it is possible to transplant the patient's cells (autologous) following gene transfer with viral vectors. The second strategy is preferable since it increases the chances and percentage of engraftment with reduced acute side effects because of a reduced pre‐conditioning treatment required and no need for immunosuppressive pharmacological treatment [87]. However, the efficiency of HSPC engraftment depends on the pre‐conditioning regimens of the host. Chemo‐ and/or radiotherapy is required before transplantation to create space for transplanted cells in the hematopoietic niche in the bone marrow [88], but preconditioning regimes represent an open issue for cell therapeutic applications, and recipients, including hemophilic patients, are generally more sensitive to adverse events caused by these treatments. More recent clinical studies are evaluating the possibility to mitigate the side effects of preconditioning by mobilization‐based and/or antibody‐based conditioning (e.g., NCT05357482).

One of the primary concerns associated with LV arises from their integrating nature, which poses risks of genotoxicity and insertional mutagenesis due to random integrations. These risks are particularly significant considering the adverse effects observed in clinical trials involving γ‐retroviral vector (RV)‐based ex vivo gene therapy [89, 90, 91]. However, LV have demonstrated superior performance in HSPC compared to RV as reported by recent updates from clinical studies follow ups [37, 38, 92, 93, 94, 95].

AAV are the preferred viral vectors for the in vivo treatment of genetic diseases due to their ability to transduce both senescent and proliferating cells, especially because they are not integrating vectors but remain in long‐term episomal form in the transduced cells/tissues. However, despite their non‐integrative nature, they show a low level of integration, the percentage of which depends on the serotype used, the target tissue and the dose administered [96]. Previous preclinical studies have demonstrated that AAV are capable of integration with evidence of genotoxicity in neonatal mice [97, 98]. These studies showed that the age of treatment is an important key point, especially in the occurrence of AAV‐mediated insertional mutagenesis observed in neonatal mice, while studies with older (juvenile/adult) mice were not able to demonstrate AAV‐induced tumorigenesis or to correlate the increased incidence of tumor formation in treated mice to AAV‐mediated insertional mutagenesis [99, 100]. However, long‐term studies (6–10 years) in larger animal models, such as dogs and non‐human primates, following administration of AAV‐FIX or AAV‐FVIII did not show tumor formation or alteration of hepatic functions [101, 102]. In addition, the administration of high doses of AAV has been studied in clinical trials and to date, conversely to what has been observed for murine models, no cases of tumorigenesis have been reported [96].

Additional data related to AAV integration rate are required to exclude the risk of insertional mutagenesis in humans, since to date little is known related to the AAV integration sites following hemophilia gene therapy in clinical trials, mainly due to the invasiveness of biopsy procedures.

The concerns surrounding genome editing for hemophilia gene therapy largely mirror those associated with viral vector‐based delivery systems. While AAV vectors are highly efficient in delivering the CRISPR‐Cas9 machinery, their prolonged expression of the Cas9 protein increases the risk of off‐target DNA cleavage, potentially leading to genetic damage and other adverse effects [103]. To mitigate this risk, the delivery of Cas9 nuclease in the form of mRNA with LNPs is a safer alternative since with this approach the nuclease's activity lasts for a shorter period, reducing the risk of off‐target events. Moreover, mRNA‐based delivery elicits a weaker immune response compared to the bacterial Cas9 protein, further enhancing its safety profile [104].

Nuclease‐based gene editing approaches, which exploit homologous recombination (HR), hold great promise for treating blood disorders. However, they present low efficiency and safety risks due to the possibility of DNA breaks at unwanted locations, called off‐target double strand breaks (DSBs) [105]. Newer DNA editing technologies, such as base editing (BE) and prime editing (PE), offer a very promising approach to permanently and efficiently correct point mutations. Unlike older methods, these new strategies do not rely on DNA double‐strand breaks (DSBs) or homologous recombination (HR), making these methods safer and more effective, with a lower risk of adverse effects [106]. Despite the promising results of recent preclinical studies in hemophilia using these approaches [107, 108, 109], these gene‐editing therapies are not yet ready for clinical use. Nonetheless, their potential for a permanent cure is very high, making it one of the most promising frontiers in hemophilia treatment.

## 5 Concluding Remarks

Gene therapy for hemophilia is a relatively young yet rapidly advancing field that in the last 15 years has made substantial progress, demonstrating its feasibility to treat both HA and HB.

However, despite the encouraging results shown by the follow up of patients treated with ROCTAVIAN and HEMGENIX, the field likewise gene therapy in general requires further data collection to expand clinical experience and continues to offer opportunities for improvements.

Ensuring durable efficacy and safety requires more comprehensive clinical trial data to deepen our understanding of long‐term outcomes. While current results are encouraging, potential limitations—such as those observed in preclinical models—must be carefully considered. This underscores the importance of extended follow‐up studies to assess the persistence of gene expression and to identify, mitigate, and prevent adverse effects over time.

Despite existing limitations, ongoing research aimed at refining gene transfer platforms and therapeutic strategies is expected to enhance the stability, safety, and reliability of hemophilia gene therapy. These advancements bring the field closer to realizing its goal: a one‐time treatment capable of delivering lifelong benefits to patients.

## Funding

This work was supported by CSP—Compagnia San Paolo Trapezio (68155) and PNRR MUR—M4C2—CN RNA & GENE THERAPY—Spoke 1, ALLEVIATE.

## Conflicts of Interest

The authors declare no conflicts of interest.
